# The Nucleotide Exchange Factor Ric-8A Is a Chaperone for the Conformationally Dynamic Nucleotide-Free State of Gαi1

**DOI:** 10.1371/journal.pone.0023197

**Published:** 2011-08-11

**Authors:** Celestine J. Thomas, Klára Briknarová, Jonathan K. Hilmer, Navid Movahed, Brian Bothner, John P. Sumida, Gregory G. Tall, Stephen R. Sprang

**Affiliations:** 1 Center for Biomolecular Structure and Dynamics, The University of Montana, Missoula, Montana, United States of America; 2 Division of Biological Science, The University of Montana, Missoula, Montana, United States of America; 3 Department of Chemistry and Biochemistry, The University of Montana, Missoula, Montana, United States of America; 4 Proteomics and Mass Spectrometry Facility, Department of Chemistry and Biochemistry, Montana State University, Bozeman, Montana, United States of America; 5 Bioanalytical Pharmacy Core, University of Washington, Seattle, Washington, United States of America; 6 Department of Pharmacology and Physiology, School of Medicine and Dentistry, University of Rochester Medical Center, Rochester, New York, United States of America; University of Queensland, Australia

## Abstract

Heterotrimeric G protein α subunits are activated upon exchange of GDP for GTP at the nucleotide binding site of Gα, catalyzed by guanine nucleotide exchange factors (GEFs). In addition to transmembrane G protein-coupled receptors (GPCRs), which act on G protein heterotrimers, members of the family cytosolic proteins typified by mammalian Ric-8A are GEFs for Gi/q/12/13-class Gα subunits. Ric-8A binds to Gα•GDP, resulting in the release of GDP. The Ric-8A complex with nucleotide-free Gαi1 is stable, but dissociates upon binding of GTP to Gαi1. To gain insight into the mechanism of Ric-8A-catalyzed GDP release from Gαi1, experiments were conducted to characterize the physical state of nucleotide-free Gαi1 (hereafter referred to as Gαi1[ ]) in solution, both as a monomeric species, and in the complex with Ric-8A. We found that Ric-8A-bound, nucleotide-free Gαi1 is more accessible to trypsinolysis than Gαi1•GDP, but less so than Gαi1[ ] alone. The TROSY-HSQC spectrum of [^15^N]Gαi1[ ] bound to Ric-8A shows considerable loss of peak intensity relative to that of [^15^N]Gαi1•GDP. Hydrogen-deuterium exchange in Gαi1[ ] bound to Ric-8A is 1.5-fold more extensive than in Gαi1•GDP. Differential scanning calorimetry shows that both Ric-8A and Gαi1•GDP undergo cooperative, irreversible unfolding transitions at 47° and 52°, respectively, while nucleotide-free Gαi1 shows a broad, weak transition near 35°. The unfolding transition for Ric-8A:Gαi1[ ] is complex, with a broad transition that peaks at 50°, suggesting that both Ric-8A and Gαi1[ ] are stabilized within the complex, relative to their respective free states. The C-terminus of Gαi1 is shown to be a critical binding element for Ric-8A, as is also the case for GPCRs, suggesting that the two types of GEF might promote nucleotide exchange by similar mechanisms, by acting as chaperones for the unstable and dynamic nucleotide-free state of Gα.

## Introduction

As members of the Ras superfamily of regulatory GTP binding proteins, heterotrimeric G protein α subunits (Gα) undergo cycles of activation and deactivation driven by binding and hydrolysis of GTP [1]. Conversion to the basal, inactive state results from the intrinsic GTP hydrolyase activity of the G protein. Reactivation is achieved by replacement of GDP by GTP at the nucleotide binding site, catalyzed by guanine nucleotide exchange factors (GEFs). Although the structural events that accompany GEF-catalyzed nucleotide exchange on small, Ras-like G proteins are relatively well understood [2], the mechanism of heterotrimeric G protein activation remains enigmatic. Agonist-activated, transmembrane G protein-coupled receptors (GPCRs) [3] are the best characterized heterotrimeric G protein GEFs. GPCRs act on plasma membrane-localized G protein heterotrimers that consist of GDP-bound Gα tightly associated with heterodimers of Gβ and Gγ subunits. Recently, members of a family of predominantly cytosolic proteins, typified by mammalian Ric-8A, were identified as non-receptor GEFs that catalyze nucleotide exchange directly on Gα subunits of the Gi/o/q/12/13 families [4]. Across phylogeny, Ric-8A paralogs act in GPCR-independent pathways to orient mitotic spindles in asymmetric cell division, as demonstrated in (*C. elegans* [5], [6], *Drosophila* [7], and mammalian cells [8].

Ric-8A is a soluble 59.7 kDa protein predicted to adopt a superhelical structure composed of α-helical armadillo repeats [9]. In contrast to GPCRs, Ric-8A catalyzes the release of GDP directly on Gα subunits, but has markedly weak affinity for Gα bound to GTP or non-hydrolyzable GTP analogs [4]. Upon binding to Gαi1•GDP, Ric-8A catalyzes GDP release and forms a stable nucleotide-free Ric-8A:Gαi1[ ] complex (empty brackets:“[ ]”, denote absence of bound nucleotide). In the presence of GTP, the complex dissociates to yield free Ric-8A and Gαi1•GTP [4].

Using limited proteolysis, circular dichroism (CD) spectroscopy, heteronuclear NMR spectroscopy, hydrogen-deuterium exchange mass spectrometry (HD-MS), and differential scanning calorimetry (DSC), we have found that Ric-8A stabilizes Gαi1 in a conformationally dynamic and heterogeneous state which, we propose, facilitates GDP release and subsequent GTP binding. We show that the C-terminus of Gαi1 is a critical binding element for Ric-8A recognition and activity, as is also the case for GPCRs [10], [11], suggesting that the two GEFs may act by convergent mechanisms.

## Results

### The smallest fragment of Ric-8A with full GEF activity encompasses most of the protein

**Figure 1:**
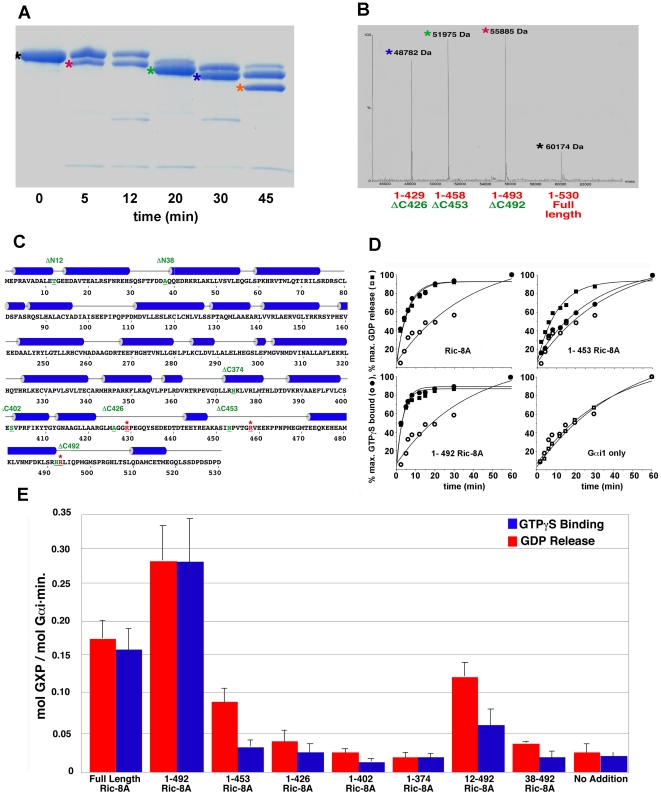
GEF activity of purified Ric-8A fragments defined by limited trypsinolysis and secondary structure analysis. (A) Coomassie-stained SDS PAGE analysis of Ric-8A after trypsinization for the times indicated below each lane; unique fragments are identified by colored asterisks. (B) Electrospray mass spectrometric analysis of Ric-8A tryptic digest fragments extracted from the SDS PAGE gel shown in panel A; peaks identified by asterisks refer to corresponding bands shown in panel A. Fragment masses (Da) are indicated at each peak position. (C) Amino acid sequence of rat Ric-8A; cylinders indicate helical segments predicted using JPRED [51]. Residue codes colored red indicate sites of proteolytic cleavage (see panel A). Residue codes in green indicate N or C-termini of recombinant Ric-8A fragments engineered to coincide approximately with proteolytic sites or predicted secondary structure boundaries: ΔC492 denotes the Ric-8A fragment comprising residues 1–492. Both N-terminal truncations ΔN12 and ΔN38 were also C-terminally truncated at residue 492 and comprised residues 12–492 and 38–492, respectively. (D) Kinetics of intrinsic (open symbols) or Ric-8A-stimulated (filled symbols) GDP release (squares) from, or GTPγ binding to (circles) myristoylated Gαi1 were determined by a filter binding assay using radiolabeled nucleotides as described [4]. Upper left panel, Gαi1 (200 nM) nucleotide binding and release in the presence of full-length Ric-8A (200 nM); lower left panel, ΔC492Ric-8A (200 nM); upper right panel, ΔC453Ric-8A (200 nM); lower right panel, Gαi1 alone. Data for each panel are normalized to maximum GDP released or GTPγS bound in a single experiment. Data points represent the average of three experiments; standard deviation from the mean is <10%. Time course of GTPγS binding in the absence of Ric-8A, shown at lower right, is replicated in the other panels for comparison. (E), Histogram showing relative rates of Gαi1 GDP release (red bars) and GTPγS binding (blue bars) catalyzed by Ric-8A and Ric-8A truncation mutants (200 nM). Error bars represent +/− one standard deviation of the apparent first-order rate constants determined in three replicates.

**Figure 2:**
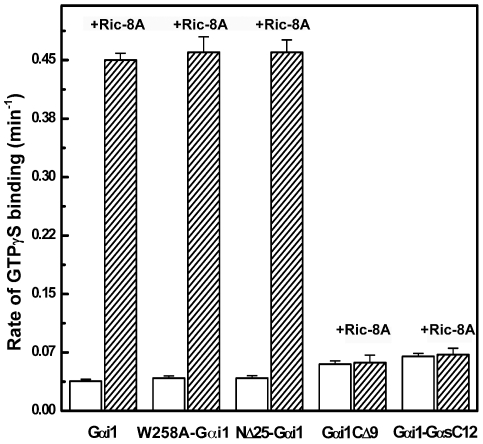
Intrinsic and Ric-8A-catalyzed GTPγS binding rates of the Gαi1 proteins used in this study. Intrinsic and Ric-8A-catalyzed kinetics of binding of GTPγS to wild-type Gαi1, W258A-Gαi1, NΔ25Gαi1, GαiCΔ9 and Gαi1-GαsC12 were measured using a fluorescence binding assay [12], [47]. 400 µl of protein (1 µM) in the GDP bound form was equilibrated for 10–15 min at 25°C in a cuvette. A 10-fold excess of GTPγS was added and fluorescence at 340 nm upon excitation at 290 nm was monitored in the absence (open bars) or presence (filled bars) of Ric-8A (1 µM). Error bars represent +/− one standard deviation apparent first-order rate constants determined in three replicates.

We conducted limited trypsin proteolysis, together with mass spectroscopic and secondary structural analysis, to define a minimal fragment of Ric-8A that retained the activity of the full-length protein (Fig. 1A–C). The fragment encompassing residues 1–492 (Ric-8AΔC492) exceeded full-length Ric-8A in GEF activity, whereas C-terminal truncations of successive predicted helical regions (ΔC426, ΔC453) or truncation of the N-terminus (ΔN12, ΔN38) in the background of ΔC492 retained GDP release activity that was uncoupled from GTPγS binding stimulatory activity (Fig. 1D,E). Truncated proteins (ΔC402, ΔC374) bound Gαi1•GDP weakly (data not shown) but had no nucleotide release or GEF activity. Because it is both more abundantly expressed in *Escherischia coli* and appears to biochemically more stable as well as more active than the full-length protein, we chose to conduct subsequent experiments with Ric-8AΔC492. To enhance the sensitivity of tryptophan fluorescence assays of GEF activity, we used a non-myristoylated Gαi1 mutant in which Trp 258 was substituted with alanine. The W258A mutation did not impair GTP binding, GTPase activity, or susceptibility to the GEF activity of Ric-8A (Fig. 2) [12]. For brevity, we refer to ΔC492Ric-8A and W258AGαi1 as Ric-8A and Gαi1, respectively.

### Relative to Gαi1•GDP, nucleotide-free Gαi1 is more accessible to protease digestion, and deficient in secondary structure

**Figure 3:**
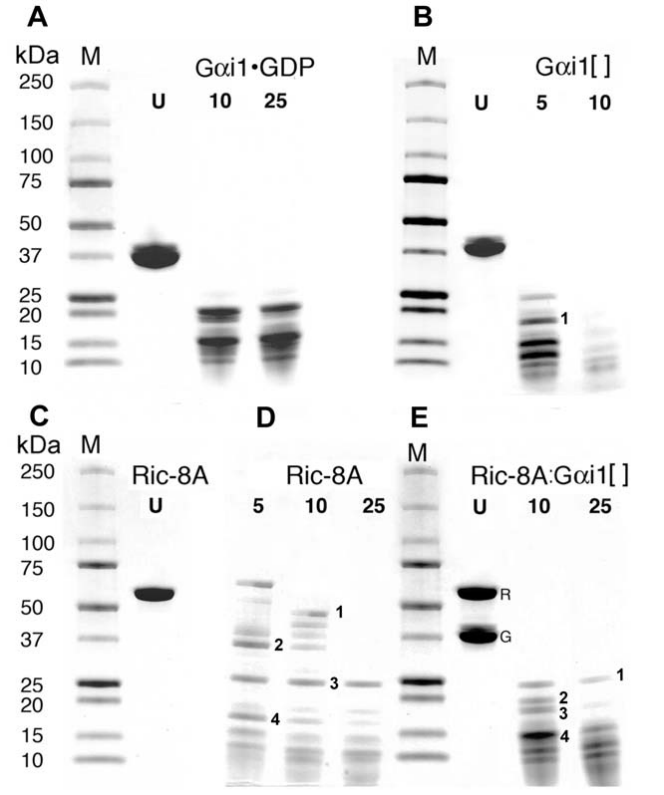
Ric-8A provides limited protection of nucleotide-free Gαi1 from trypsin digestion. Samples were incubated with TPCK treated trypsin at a 1∶1000 molar ratio (trypsin∶sample) at 4°C, withdrawn at the indicated time points, separated by SDS-PAGE and visualized by Coomassie blue staining. (A) Gαi1•GDP: lanes from left to right: molecular weight markers, M; untreated Gαi1•GDP, U; samples digested for 10 and 25 minutes. (B) nucleotide-free Gαi1[ ]: markers, M; untreated Gαi1[ ], U; samples digested for 5 and 10 minutes. Mass spectroscopic analysis identifies band 1 as Gαi1 residues 21–179: observed/calculated mass 17,761/17,774 Da. (C) Ric-8A: markers, M; untreated, U. (D) Ric-8A: after 5, 10 and 15 minutes of trypsin digestion. Mass analysis identifies band 1 as Ric-8A residues 1–408: 46,218/46,207 Da; band 2, residues 72–378: 34,815/34,799; band 3, residues 141–348: 23,834/23,804 Da; band 4, residues 62–178: 13,563/13,523 Da. (E) Ric-8A: Gαi1[ ] complex: markers, M; untreated Ric-8A:Gαi1[ ] complex; R and G indicate bands for intact Ric-8A and Gαi1, respectively; Ric-8A:Gαi1[ ] complex digested for 10 and 25 minutes. Mass analysis identifies band 1 as Ric-8A residues 141–348: 23,834/23,804 Da (present also as band 3 in Panel D); band 2, Gαi1 residues 17–191: 19,646/19,652 Da; band 3, Gαi1 residues 21–179: 17,753/17,761 Da (present as band 1 in panel B); band 4: Gαi1 residues 10–141: 14,532/14,520 Da.

**Figure 4:**
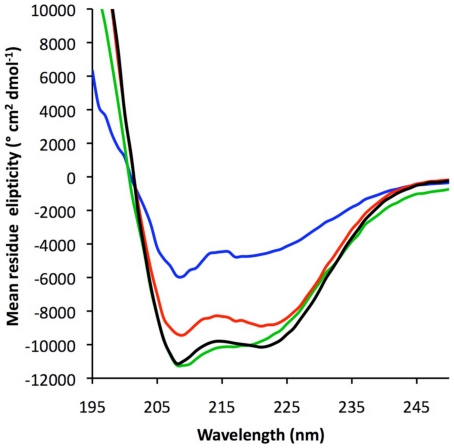
Nucleotide-free Gαi1 is relatively unstructured in comparison to Gαi1•GDP, but regains helical secondary structure in the complex with Ric-8A. Circular dichroic spectra were normalized as mean residue elipticity, and predicted secondary structure assignments are: Ric-8A, *red*: 87% α-helix; Ric-8A:Gαi1[ ], *black*: 87% α-helix, 0.5% β-strand; Gαi1•GDP, *green*: 51% α-helix, and 11% β-strand; Gαi1[ ], *blue*, 38% α-helix, 9% β-strand.

Trypsinolysis experiments demonstrated that Gαi1[ ] was substantially more protease-sensitive than Gαi1•GDP (Figures 3A and 3B) and was more rapidly degraded into <20 kDa fragments. The distribution of cleavage products is different in the free and GDP-bound states. Normalized as mean residue elipticity, the CD spectrum of Gαi1[ ] showed an overall reduction of regular secondary structure relative to Gαi1•GDP (Figure 4). These results accord with earlier findings that Gαi1[ ] is converted into a misfolded species with low affinity for guanine nucleotides [13]. Gαi1[ ] bound to Ric-8A was more resistant to trypsinolysis than free Gαi1[ ], as indicated by the persistence of fragments labeled 2 though 4 at the 10 minute time point in Figure 3E. Note, for example, that band 1, visible at the 5 minute time point of Gαi1[ ], is degraded after 10 minutes of protease exposure (Figure 3B). The same fragment persisted after 10 minutes in the complex with Ric-8A (Figure 3E, band 3). Fragments 2–4 encompass the N-terminal residues of the Ras domain beyond the P-loop, together with most or all of the helical domain of Gαi1 [14]. Nevertheless, Ric-8A-bound Gαi1[ ] was still more susceptible to proteolysis than Gαi1•GDP (Figure 3A). In contrast, if bound to Gαi1, Ric-8A was more sensitive to protease digestion than free Ric-8A (Figures 3D and 3E). After 10 minutes of protease digestion of Ric-8A:Gαi1[ ], all Ric-8A fragments with molecular weights greater than ∼24 KDa were degraded, yet several fragments of greater length remained intact after free Ric-8A was exposed to trypsin for the same duration. For both free and Gαi1-bound Ric-8A, residues 141–348 appears to constitute a relatively protease-resistant protein core (bands 3 and 1 in Figure 3D and 3E, respectively; for reference to the predicted secondary structure of Ric-8A see Figure 1C). Note that no protease inhibitors were present in the trypsin preparation used to generate the data shown in Figure 3, so the extent of Ric-8A degradation is greater than that shown in Figure 1A.

The mass-normalized CD spectra of Ric-8A and Ric-8A:Gαi1[ ] show similar degrees of secondary structure formation. Therefore, we infer that Ric-8A-bound Gαi1[ ] possesses higher secondary structure content than free Gαi1[ ]. Both spectra are indicative of predominantly α-helical structure, whereas Gαi1•GDP shows evidence of both α-helical and α-sheet structure, which is characteristic of the Ras-like domain of this and other G proteins [1] (Figure 4). The near absence of β-sheet structure estimated from the CD spectrum of Ric-8A:Gαi1[ ] suggests that changes in secondary structure may occur in the α/β Ras-like domain of Gαi1 upon binding to Ric-8A and subsequent release of GDP.

### Peaks in the ^15^N-^1^H HSQC spectrum of Gαi1 are severely attenuated upon binding to Ric-8A

**Figure 5:**
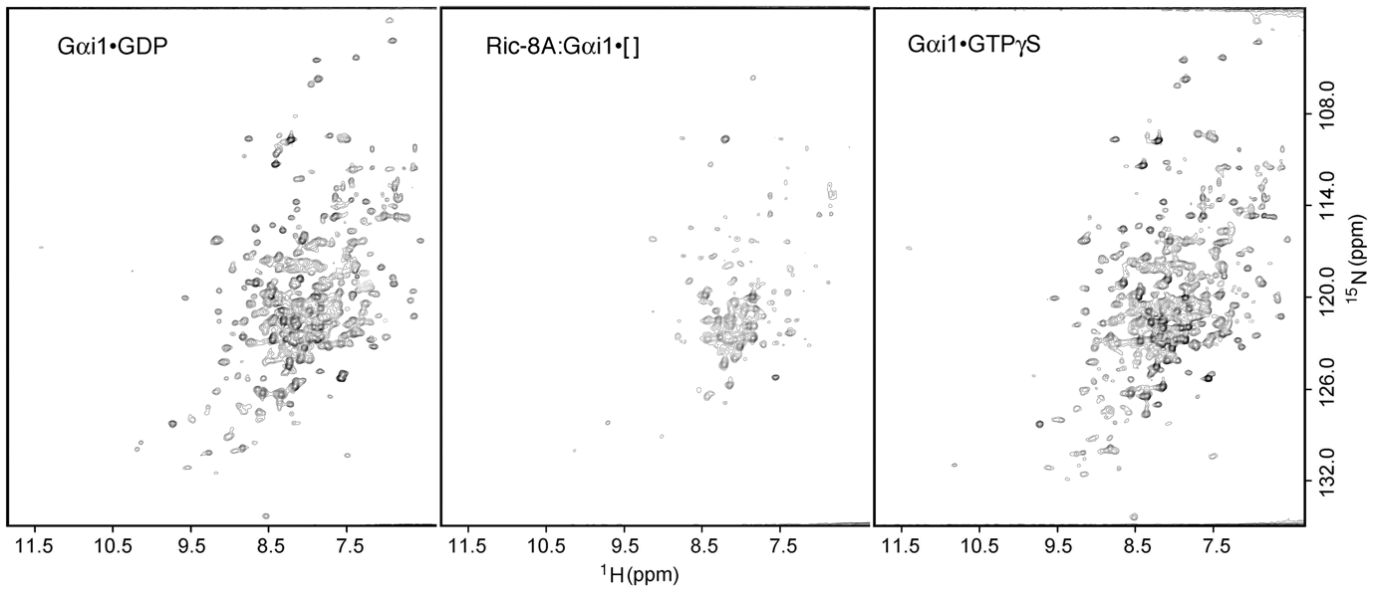
^1^H-^15^N TROSY-HSQC spectrum of Gαi1 shows extensive peak broadening and intensity loss upon binding of Ric-8A. (A) ^1^H-^15^N HSQC-TROSY spectra were acquired for [^15^N]Gαi1•GDP, and (B) Ric8A:[^15^N]Gαi1[ ]. (C) After acquisition of the Ric-8A:[^15^N]Gαi1[ ] spectrum, a five molar excess of GTPγS was added to induce dissociation of Ric-8A and formation of Gαi1•GTPγS. After a short incubation, the free Ric-8A was removed by adsorption to IMAC resin, and the ^1^H-^15^N TROSY-HSQC spectrum of the sample was recorded. Protein concentration, acquisition and processing parameters and contour levels in all panels are the same.

To elucidate the structural properties of Gαi1 bound either to nucleotides or to Ric-8A, we acquired ^1^H-^15^N Transverse Relaxation Optimized (TROSY) Heteronuclear Single Quantum Coherence (HSQC) spectra [15] of [^15^N]Gαi1. The ^1^H-^15^N TROSY-HSQC spectrum of Gαi1•GDP (Figure 5A) and Gαi1•GTPγS (data not shown) showed ∼300 moderately well resolved and dispersed peaks, comparable in quality to spectra reported by Abdulaev, *et al.* [16] for a GDP-bound chimera (Gαt/i) of transducin α (Gαt) and Gαi1. In contrast, the spectrum of [^15^N]Gαi1:Ric-8A showed considerable diminution in the amplitude of many peaks, indicative of extensive line broadening (Figure 5B). Significant changes in chemical shift were not observed upon binding to Ric-8A. Gel filtration of the sample after data collection confirmed that Ric-8A:[^15^N]Gαi1[ ] remained a homogeneous heterodimer over the time-scale of the NMR experiment, and did not aggregate (data not shown). Peak broadening observed in the spectrum of Ric-8A:[^15^N]Gαi1[ ] could result from exchange among conformational states of Ric-8A-bound Gαi1[ ] in the intermediate µs-ms time scale, and/or from slow tumbling of the 96 kDa complex. Subsequent re-acquisition of the ^1^H-^15^N TROSY-HSQC spectrum after addition of GTPγS to dissociate the complex and removal of free Ric-8A, afforded a [^15^N]Gαi1•GTPγS spectrum identical to that of Gαi1•GTPγS prepared in the absence of Ric-8A (Figure 5C), thus demonstrating that Gαi1 bound to Ric-8A in the NMR sample retained biochemical activity.

### The population of rapidly exchanging Gαi1 protons doubles upon Ric-8A binding and release of GDP

**Figure 6:**
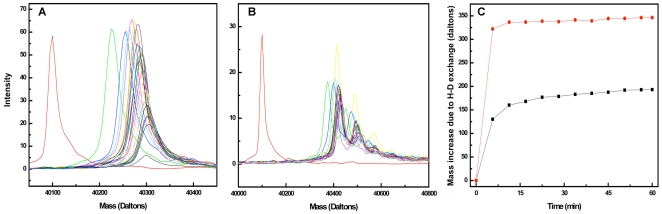
Nucleotide-free Gαi1 bound to Ric-8A exhibits rapid hydrogen/deuterium exchange kinetics relative to the Gαi1•GDP complex. (A) Mass distribution for Gαi1•GDP measured at fixed time points (see panel C) after dilution into D_2_O; the mass distribution at the zero time point, before exchange was initiated, corresponds to the red peak centered at 40.1 kDa. The average of the Gαi1 mass distribution increases as H/D exchange reaction proceeds. (B) Mass distribution for Gαi1[ ] in complex with Ric-8A measured at fixed time points after dilution into D_2_O; note that the Gαi1[ ] mass distribution becomes multimodal as the H/D exchange reaction proceeds. (C) The increase in mass (Da), determined at the centroid of the mass distribution of Gαi1•GDP (black squares) and Gαi1[ ] derived from the complex with Ric-8A (red circles) is plotted as a function of time after rapid dilution from aqueous buffer into D_2_O.

To test the hypothesis that Ric-8A-bound Gαi1[ ] adopts a state of high conformational flexibility, we conducted HD-MS exchange experiments [17] to directly assess changes in structural dynamics of Gαi1 upon formation of the Ric-8A:Gαi1[ ] complex. Hydrogen-deuterium exchange in either Gαi1•GDP or Ric-8A:Gαi1[ ] was initiated by rapid dilution of the proteins into D_2_O buffer. To determine the rate and extent of HD exchange, the exchange reaction was quenched with formic acid/acetonitrile at successive time intervals, and the products analyzed by electrospray mass spectrometry (ES-MS). For each time-point, the mass distribution of Gαi1 was determined by deconvolution of the raw m/z spectrum (Figure 6A,B). The mass distribution of Gαi1•GDP remained unimodal throughout the 60 minute exchange period (Figure 6A), whereas that for Gαi1[ ] bound to Ric-8A evolved into a multimodal distribution, suggestive of conformational heterogeneity (Figure 6B). Analysis of these data revealed a nearly four-fold greater initial rate of deuterium exchange in Ric-8A-bound Gαi1[ ] than in Gαi1•GDP (Figure 6C). After 60 minutes of exposure to D_2_O, the mass of Gαi1[ ] in the complex with Ric-8A increased by ∼340 Da, accounting for more than half of all the exchangeable Gαi1 protons, *versus* a ∼210 Da mass increase in Gαi1•GDP alone. The enhanced rate and extent of deuterium substitution is indicative of greater solvent accessibility at exchangeable sites in Ric-8A-bound Gαi1 than in Gαi1•GDP, most likely due to amplified breathing motions in the Ric-8A:Gαi1[ ] complex [18].

### Thermodynamic stability of both nucleotide-free Gαi1 and Ric-8A increase upon complex formation

We used differential scanning calorimetry (DSC), by which change in heat capacity (Cp) is measured as a function of temperature, to determine the modality and mid-point temperatures (T_m_) for the unfolding transitions of Gαi1•GDP, Gαi1[ ], Ric-8A and Ric-8A:Gαi1[ ] [19]. At temperatures below and above the thermal unfolding transition of a protein, the Cp exhibits a linear, typically positive, dependence on the temperature of the native and unfolded states, respectively. In the region of the thermal transition, Cp exceeds that of both the denatured and native states as hydrophobic groups are increasingly exposed to the aqueous solvent, and reaches a maximum value at T_m_ [20], [21], [22].

**Figure 7:**
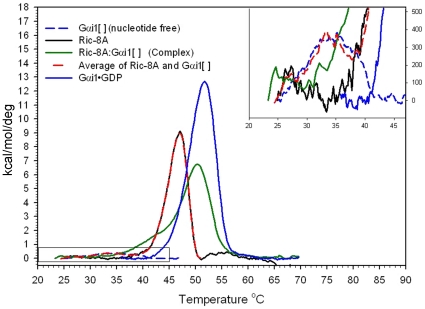
Thermal denaturation properties of Gαi1 and Ric-8A are affected by their mutual interaction. Temperature-dependence of heat capacity was measured by differential scanning calorimetry. Buffer baseline-corrected thermograms were recorded for Ric-8A (black trace), Gαi1[ ] (dashed blue trace), Gαi1•GDP (blue trace) and Ric-8A:Gαi1[ ] (green trace). The weighted average of the thermograms for Ric-8A and Gαi1[ ] (red dashed line) overlaps that of Gαi1[ ] in the temperature range below ∼37°C and that of Ric-8A above that temperature, and is distinct from the thermogram of Ric-8A:Gαi1[ ]. The inset shows a magnified view of the four thermograms and the weighted average function in the 20°C–45°C range.

Gαi1•GDP underwent an irreversible cooperative unfolding transition with T_m_ = 52°C (Figure 7, blue trace). The irreversible nature of the transition of this and the other proteins and protein complexes reported here precludes accurate determination of the enthalpy of unfolding, but allows comparison of the significant thermal features of the four species when measured at equivalent scan rates. Only a weak transition near 33°C was observed for Gαi1[ ] (Figure 7, dashed blue trace), which exhibited changes in Cp that were close to the detection limits of the instrument. The nucleotide-free protein thus appears to be conformationally heterogeneous or disordered [23], consistent with its high protease sensitivity (Figure 3B) and CD spectrum (Figure 4). Ric-8A itself underwent an irreversible cooperative folding transition with T_m_ = 47°C (Figure 7, black trace). Thermal denaturation of Ric-8A:Gαi1[ ] was characterized by a nearly linear increase in heat capacity, suggestive of non-cooperative unfolding, followed by a discrete cooperative transition at 50°C (Figure 7, green trace), higher than the melting temperature of free Ric-8A. This latter T_m_ is invariant with protein concentration (data not shown), indicating that the complex remains intact throughout the transition. The denaturation profile of Ric-8A:Gαi1[ ] complex cannot be modeled as weighted average of the profiles of Ric-8A and Gαi1[ ] (Figure 7, red dashed line), hence the complex has unique thermodynamic properties relative to Gαi1[ ] and Ric-8A.

### The C-terminus of Gαi1 is a specific and critical recognition element for Ric-8A binding and GEF activity

**Figure 8:**
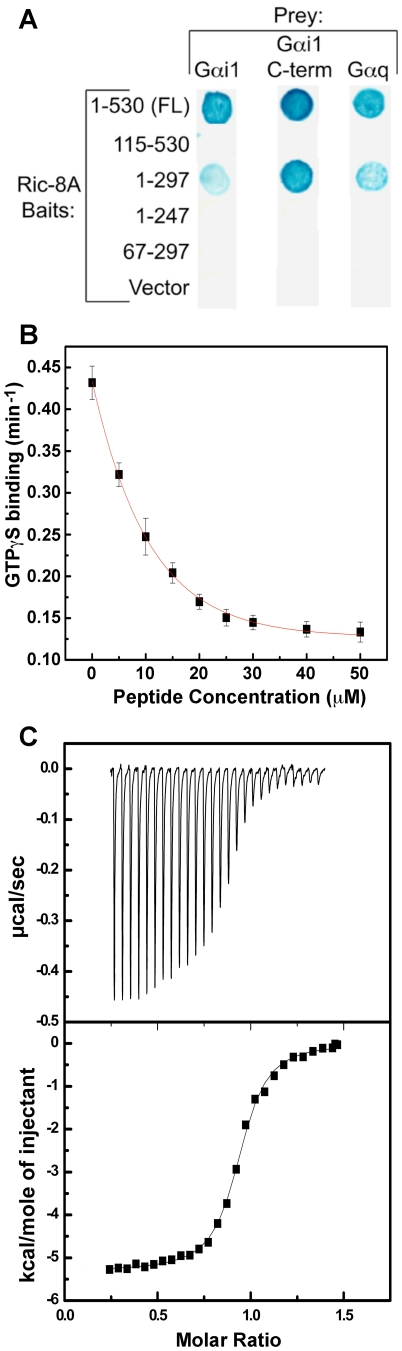
The carboxyl-terminus of Gαi1 interacts with the amino-terminus of Ric-8A. (A) Yeast two-hybrid screen; prey clones encoding full-length Gαi1, Gαq and the carboxyl-terminal 81 amino acids of Gαi1 were tested pair-wise using a β-galactosidase filter assay for interactions with the full-length Ric-8A bait (amino acids, 1–530 FL), and truncated Ric-8A bait constructs (amino acids: 115–530, 1–297, 1–247, 67–297), and the empty prey vector. (B) Inhibition by Gαi1C18, a peptide composed of the 18 C-terminal residues of Gαi1, on Ric-8A-catalyzed binding of GTPγS to Gαi1 was assayed by fluorescence emission from an endogenous Gαi1 tryptophan. To 1 µM Gαi1•GDP in the presence of 1 µM Ric-8A, pre-incubated with different concentrations of Gαi1C18, was was added a 10-fold excess of GTPγS and fluorescence at 340 nm upon excitation at 290 nm was monitored. GTPγS binding rates, obtained by fitting the time-dependent rise in fluorescence, plotted as a function of peptide concentration, yields an IC_50_ of 23 µM for Gαi1C18 inhibition of Ric-8A nucleotide exchange activity. Data points shown are the mean, and error bars indicate the standard deviation, for three separate determinations. (C) Isothermal titration calorimetric determination of binding of Gαi1C18 to Ric-8A. Peptide (250 µM) was injected into a calorimeter cell containing 25 µM Ric-8A. The top panel shows the baseline corrected isotherm and the bottom panel indicates the fit of the same performed with a single binding site model, yielding a dissociation constant of 12 µM for binding of Gαi1C18 to Ric-8A at 25°C. The calculated enthalpy for the reaction was −5.3 kcal/mol with a stoichiometry (N) of 0.9.

The mechanism by which Ric-8A catalyzes nucleotide exchange is similar in some respects to the analogous reaction catalyzed by GPCR at Gβγ-bound Gα subunits. Experimental evidence indicates that specific recognition and binding of the C-terminus of Gα is crucial to the action of Ric-8A, just as it is for GPCRs [10], [11]. First, a yeast two-hybrid screen of a rat brain library using a bait construct comprising Ric-8A residues 1–297 yielded a prey clone expressing the C-terminal 81 residues of Gαi1, that also interacted with a full-length Ric-8A bait construct (Figure 8A). Second, the peptide Gαi1C18, which is composed of a sequence of amino acid residues identical to that of the 18 C-terminal residues of Gαi1, inhibited Ric-8A-catalyzed exchange of GTPγS for GDP with an IC_50_ of 23 µM (Figure 8B). Isothermal calorimetric measurements indicated that Gαi1C18 binds directly to Ric-8A with a K_d_ of 12 µM (Figure 8C). Third, Gαi1CΔ9, a Gαi1 truncation mutant lacking the nine C-terminal residues of the native protein, fails to serve as a substrate for Ric-8A although it retains GTP binding and hydrolytic activity [24] (Figure 2). Finally, substitution of the C-terminal twelve residues of Gαi1 with the corresponding residues of Gαs, a Gα protein that does not bind to Ric-8A [4], abrogated susceptibility to the GEF activity of Ric-8A (Figure 2) but did not impair GTP binding activity. These results are in accord with recent findings that pertussis toxin-catalyzed ADP ribosylation at the C-terminus of Gαi1 [8] and that truncation of the twelve Gαi1 C-terminal residues [25] blocks Ric-8A binding and GEF activity. On the other hand, truncation of 25 residues from the N-terminus of Gαi1 did not affect its susceptibility to the GEF activity of Ric-8A (Figure 2).

## Discussion

The experiments described in this report provide insight into the mechanism of Ric-8A-catalyzed exchange of GDP for GTP on Gαi1. In this reaction, Ric-8A:Gαi1[ ] is a stable intermediate that does not readily dissociate in the absence of GTP or non-hydrolysable GTP analogs. We have shown that, within this complex, Gαi1[ ] adopts a considerably more dynamic conformation than nucleotide-bound Gαi1, but is more structured and less susceptible to proteolysis than free Gαi1[ ]. This is in sharp contrast to most nucleotide-free complexes of small G proteins with cognate GEFs, in which both the GEF and G protein components are typically well ordered structures [2].

Ric-8A-catalyzed nucleotide exchange proceeds through a stable (in the absence of GTP) but loosely structured intermediate. Examples of enzymes that stabilize proteins in unfolded or disordered states include chaperones such as GroEL [26], and AAA+ ATPase unfoldases of ClpXP proteases and related proteases that degrade mis-folded proteins [27]. However, Ric-8A functions differently from these, in that it is not coupled to an exergonic reaction (*e.g.* ATPase activity), but does exhibit high substrate specificity and catalyzes a discrete chemical transformation. We propose that the catalytic power of Ric-8A derives, in part, from its ability to act (in rough analogy with GroEL and other unfoldases) as a chaperone for a partially unfolded or disordered conformation Gαi1[ ], thereby reducing the activation energy barrier to GDP release and GTP binding, while disfavoring unproductive side reactions that would lead to Gαi1 deactivation and aggregation. In the partially unstructured state induced and stablilized by Ric-8A, the nucleotide binding site of Gαi1 may be more solvent-accessible than in the nucleotide-bound state.

The mechanism by which Ric-8A catalyzes nucleotide exchange may be similar in some respects to the analogous reaction catalyzed by GPCR at Gβγ-bound Gα subunits. Recognition and binding of the Gα C-terminus is crucial to the action of both exchange factors. In a manner analogous to that proposed for GPCRs, Ric-8A could promote nucleotide release by gripping, and perhaps tensioning, the C-terminus of Gα and thereby weaken local tertiary structure (α5 helix, β5 and β6 strands) that is allosterically coupled to the purine binding site and switch regions [11], [28], [29]. Indeed, the substantial reduction in fluorescence emission of Trp 211 in switch II of Gαi1 upon binding to Ric-8A provides evidence for such perturbations [12]. Whether Ric-8A directly engages the switch regions of Gα is uncertain. Such interactions might be precluded since Ric-8A is able to form a transient ternary complex with Gαi1•GDP:AGS3 [12]. AGS3, a guanine nucleotide dissociation inhibitor, comprises GPR/GoLoco motifs [30], [31] that partially block the switch I/switch II interface [32]. Similarly, direct interactions between GPCRs and the switch regions of Gα are problematic on stereochemical grounds [33]. It is also noteworthy in this context that inportin-β, a protein involved in the transport of protein cargo into the nucleus, and also a presumptive structural analog of Ric-8A, has been shown to act as a GEF for Ran1•GDP [34]. Crystallographic analysis demonstrates that importin-β induces conformational changes in the switch regions of Ran1•GDP [35].

Both GPCRs and Ric-8A appear to induce or maintain a dynamic state of Gα. NMR studies of complexes between [^15^N]Gαi/t:β1γ1 and rhodopsin mimetics show that resonances in the Gα subunit are highly broadened [36], as we have observed for Ric-8A: Gαi1[ ]. In contrast, the HSQC spectrum of [^15^N]Gαi/t bound to Gβ1γ1, a complex with a molecular mass comparable to Ric-8A: [^15^N]Gαi1[ ], is well defined and similar to that of Gαi/t•GDP [37]. Recent evidence obtained from double electron electron resonance (DEER) spectroscopy shows that, in the activated rhodopsin-heterotrimer complex, nucleotide-free Gαi1 is conformationally heterogeneous, and that the Ras-like and helical domains of Gαi1, between which nucleotide is bound, swing away from each other [38]. The NMR and HD-MS data presented here suggest that nucleotide-free Gαi1 bound to Ric-8A undergoes conformational exchange, with interconversion times that are possibly in the µs-ms range. The DSC melting profile of Ric-8A:Gαi1[ ] is suggestive of a non-cooperative unfolding transition at lower temperature followed by a discrete transition near 50°C. It seems reasonable to attribute the former to a conformationally heterogeneous and dynamic Gαi1[ ] and the latter to Ric-8A, which in the complex with Gαi1[ ] is more thermostable yet also more protease accessible than unbound Ric-8A, suggesting that Ric-8A itself may undergo some structural change upon binding to Gαi1. However, structural assignment of transitions in the DSC spectra is speculative. It remains to be determined which segments of Gαi1 become mobile within the nucleotide free complex with Ric-8A, and importantly, to confirm that induction or stabilization of a partially disordered or conformationally flexible state in Gαi1 in fact reduces the kinetic energy barrier to GDP release and GTP binding.

## Materials and Methods

### Molecular Cloning and Protein Expression

The open reading frame of rat Ric-8A and truncation variants (encompassing residues 1–492, 12–492, 1–453, 1–426, 1–402, 1–374, 12–492 and 38–492) were amplified by PCR and subcloned into the pET-28a vector for expression as N-terminally hexa-histidine tagged proteins. Proteins were expressed in *Escherichia coli* BL21 (DE3)-RIPL cells in LB media containing ampicillin (120 mg/L) and induced with 300 µM isopropyl β-d-thiogalactopyranoside (IPTG) at 20°C. After overnight growth at 20°C, cells were lysed by sonication at 20°C in lysis buffer (50 mM Tris, pH 8.0, 250 mM NaCl, 2 mM DTT, and 2 mM PMSF. The cell lysate was clarified by centrifugation and loaded onto a column containing 5 ml of nickel NTA-agarose (GE Healthcare). After extensive washing with lysis buffer, proteins were eluted from the resin with buffer (50 mM Tris, pH 8.0, 150 mM NaCl and 2 mM PMSF) containing 250 mM imidazole and dialyzed in a low ionic strength buffer (50 mM Tris, pH 8.0, 2 mM DTT, and 2 mM PMSF). The dialysate was loaded onto a UNO-Q matrix (Bio-Rad) and eluted with a 0–500 mM NaCl gradient on an AKTA FPLC system (GE Healthcare). Pure Ric-8AΔC492 eluted from the matrix at 165–175 mM NaCl.

Rat Gαi1 was expressed as a tobacco etch virus protease (TEV)-cleavable, N-terminal glutathione-S-transferase (GST) fusion protein as described [12]. W258A-Gαi1, in which the tryptophan residue at position 258 is substituted by alanine, was generated by use of the QuikChange (Stratagene) kit according to the manufacturer's protocol, using the pDEST-15 vector harboring wild type GST-Gαi1 as a template. To generate NΔ25-Gαi1, from which the N-terminal 25 residues of the native protein are deleted, attB-modified primers corresponding to amino acids 25 to 35 and 343 to 353 of Gαi1 were used for PCR amplification and cloning of the fragment into the pDEST15 vector. W258-Gαi1 and NΔ25-Gαi1 were expressed and purified as described [12].

The plasmid pBN905, which expresses rat Gαi1ΔC9, lacking the C-terminal nine residues of the native protein, fused in-frame to intein-CBD cDNA in the pTXB3 expression vector (New England Biolabs), was a kind gift from Dr. T.J. Baranski, Washington University, St. Louis, MO. Gαi1ΔC9 was expressed and purified as described [39]. With the exception of experiments summarized in Figure 1, 2 and 8A, all other experiments were performed with Ric-8AΔC492 and W258A-Gαi1, which we henceforth refer to as Ric-8A and Gαi1, respectively.

Nucleotide-free Gαi1 proteins were prepared by the method of Ferguson and Higashijima [40], using exchange and dialysis buffers composed of 50 mM Tris.HCl, pH 8.1, 2 mM Tris (2-carboxyethyl)phosphene (TCEP) with 20% glycerol (v/v) and 150 mM NaCl. GTPγS-bound Gαi1 was prepared as described [41].

### Preparation of ^15^N-labeled proteins

[^15^N]Gαi1 was prepared as described with minor modifications [42]. Briefly, transformed *E. coli* cells were grown in minimal media supplemented with [^15^N]NH_4_Cl (Cambridge Isotopes, 99.8% purity) and [^15^N]Bioexpress Cell Growth media (10 ml of 10× concentrate/liter of media) (Cambridge Isotope Labs), induced with 500 µM IPTG at 20°C and allowed to express Gαi1 overnight at the same temperature. The purification protocol was identical to that used for native proteins and the yields were approximately one third lower.

### Preparation of Ric-8A:Gαi1 complexes

Nucleotide-free Ric-8A:Gαi1 and Ric-8A:[^15^N]Gαi1 complexes were generated by incubating equimolar concentrations of Ric-8A (500 µl of 150 µM protein) with Gαi1•GDP or [^15^N]Gαi1 (500 µl of 150 µM protein) overnight in sample buffer (20 mM Tris•HCl, pH 8.0, 150 mM NaCl, 2 mM DTT, and 5 mM EDTA) containing 50 µl of immobilized alkaline phosphatase (Sigma) to hydrolyze released nucleotide, and gently rocked at 4°C. The immobilized alkaline phosphatase was removed by centrifugation, and complex was gel-filtered over tandem Superdex 200/75 gel filtration columns pre-equilibrated in 20 mM Tris, pH 8.0, 150 mM NaCl, 2 mM DTT and eluted at a flow rate of 0.4 ml/min using an AKTA FPLC (GE Healthcare).

### Trypsin Protection Assays

To samples containing Ric-8A, Gαi1•GDP, Gαi1[ ] or Ric-8A:Gαi1 (50 µM in 50 mM Tris-HCl, pH 8.0, 150 mM NaCl and 2 mM DTT), L-1-*p*-tosylamido-2-phenylethyl chloromethyl ketone (TPCK)-treated trypsin (Sigma) was added at a molar ratio of 1000∶1. Samples were incubated at 4°C for 5, 10, 15 and 25 minutes. For each time point, a 10 µl aliquot was withdrawn, diluted in SDS-PAGE loading buffer, boiled, separated by SDS-PAGE and visualized by Coomassie staining. Proteolytic products were eluted from the gel slices and subjected to MALDI-TOF mass spectrometry on a Voyager DE3 (Applied Biosystems) or by electrospray mass spectometry using on a Agilent 6520 QTOF. Limited trypsinolysis for mass spectrometric identification of large Ric-8A fragments (Figure 1) was conducted in the presence of a 1∶2 molar ratio of bovine pancreatic aprotinin to TPCK-trypsin. Proteolytic products were eluted from the gel slices and subjected to electrospray mass spectrometry and N-terminal sequencing at the Protein Chemistry Core Facility of the University of Texas Southwestern Medical Center.

### Yeast two hybrid experiments

A rat brain yeast two-hybrid prey library [43] was screened with a pVJL11 [44] bait construct encoding the amino-terminal 297 amino acids of Ric-8A (1–297) in the L40 yeast strain [45] with a prey clone consisting of the carboxyl-terminal 81 amino acids of Gαi1 (Gαi1 C-term). Full-length Gαi1 and Gαq, and the Gαi1 C-term preys, in pGADGH (Clonetech) were then tested pair-wise using a β-galactosidase filter assay for interactions with the full-length Ric-8A bait (amino acids, 1–530 FL), and truncated Ric-8A bait constructs that coded for different regions of the Ric-8A protein (amino acids: 115–530, 1–297, 1–247, 67–297), or the empty prey vector.

### [^35^S]GTPγS Binding Assays

Binding of [^35^S]GTPγS to wild-type Gαi1 or W258A-Gαi1, in the presence or absence of Ric-8A was performed using a filter binding method [46] in a 50 mM Tris-HCl, pH 8.0, 150 mM NaCl, 2 mM DTT and 0.05% polyoxyethylene lauryl ether (C12E10).

### Pull-down assays for Gαi1 binding to Ric-8A fragments

Equimolar amounts of Ric-8A and Gαi1 (10 µM protein in 50 mM TRIS. HCl, pH 8.0, 150 mM NaCl, 2 mM DTT and 0.05% C12E10) were incubated overnight at 4°C. 10 µl of a 50% slurry of Ni^+2^ IMAC (BioRad) resin was then added to the mixture, incubated for one hour, washed thrice with 500 µl of wash buffer (50 mM Tris, pH 8.0, 250 mM NaCl, 1 mM DTT and 2 mM PMSF) and the proteins retained on the beads were visualized by Coomassie stained SDS-PAGE.

### Peptide synthesis

An amidated peptide, Gαi1C18, corresponding to the C-terminal 18 residues of rat Gαi1 (DAVTDVIIKNNLKDCGLF) was synthesized using standard FMOC chemistry by the Protein Chemistry Core laboratory at UT Southwestern Medical Center at Dallas and purified to near homogeneity by HPLC (Agilent Technologies) on a pre-packed C18 matrix (Waters). Mass of the peptide was confirmed by MALDI-TOF mass spectrometry (Voyager DE, Applied Biosystems).

### Peptide competition assays

Exchange of GTPγS for GDP bound to Gαi1 or Gαi1CΔ9 was followed by monitoring the change in the tryptophan fluorescence of Gαi1, as described [12]. Gαi1•GDP (1 µM) in 20 mM HEPES, pH 8.0, 100 mM NaCl, 10 mM MgCl_2_, 1 mM DTT, and 0.05% C12E10 in a reaction volume of 400 µl was allowed to equilibrate for 10–15 min at 20°C in a quartz fluorescence cuvette. GTPγS (final concentration, 10 µM) was added to the reaction mixture in the absence or presence of 1 µM Ric-8A, and the increase in fluorescence at 340 nm was monitored upon excitation at 290 nm [47]. Exchange kinetics were also measured in the presence of 1 µM Ric-8A and (5–50 µM) Gαi1C18. Protein and peptide mixtures were preincubated for one hour before addition of GTPγS. Fluorescence measurements were conducted using an LS55 spectrofluorometer (PerkinElmer Life Sciences) attached to a circulating water bath to maintain a steady sample temperature of 20°C. Excitation and emission slit widths were set at 2.5 nm. All exciting light was eliminated by use of a 290 nm cut-off filter positioned in front of the emission photomultiplier.

### Circular Dichroism spectroscopy

Gαi1•GDP, Ric-8A, Gαi1[ ] or the nucleotide free binary complex of the two proteins at 4 µM each in 25 mM HEPES, pH 7.2, 150 mM NaCl and 2 mM DTT and, in the case of Gαi1[ ], 20% v/v glycerol, were dispensed into a 300-µl quartz cuvette with a 1 mm path length. CD spectra in the range of 195–245 nm were measured at a scan rate of 1 nm/min using a PiStar-180 CD spectrometer (Applied Photophysics). The scans were repeated thrice; the data were averaged and the CD spectra of the buffer was subtracted. The optical path and the cuvette chamber were continually flushed with a nitrogen flow throughout the course of the experiment. Secondary structure analysis was performed using K2D2 [48].

### NMR spectroscopy

Protein samples for NMR spectroscopy ([^15^N]Gαi1•GDP, [^15^N]Gαi1:Ric-8A or [^15^N]Gαi1•GTPγS) were dialyzed against 20 mM sodium phosphate, pH 6.8, 75 mM NaCl and 2 mM DTT in 10% ^2^H_2_O/90% H_2_O, and concentrated to 250 µM. The [^15^N]Gαi1•GTPγS sample was prepared from the [^15^N]Gαi1:Ric-8A complex by addition of five molar excess of GTPγS and incubation for 10 minutes at 25°C. Free Ric-8A, and non-dissociated [^15^N]Gαi1:Ric-8A complex were then removed with Ni^+2^ IMAC resin (BioRad). ^1^H-^15^N TROSY-HSQC spectra [49] were acquired at 25°C on a 600 MHz Varian NMR System equipped with a salt-tolerant cold probe and processed with Felix 2004 (Felix NMR, Inc.).

### Hydrogen-Deuterium Exchange Mass Spectrometry

Hydrogen-deuterium exchange of the Gαi1•GDP or Ric-8A:Gαi1[ ] was analyzed by automated reverse-phase HPLC coupled to electrospray ionization TOF mass spectrometry. The HPLC consisted of an Agilent 1100 HPLC with a G1377a autosampler, and the ESI-TOF was a Bruker microTOF. Following initiation of the reaction by ten-fold dilution of protein stock (1 mg/ml Gαi1•GDP or Ric-8A:Gαi1[ ], in 20 mM sodium phosphate, pH 6.8, 100 mM NaCl and 1 mM DTT) into D_2_O, the reaction mixture was pipetted into a sealed autosampler vial and the autosampler was used to draw aliquots at regular time intervals. Quenching of the exchange reaction was achieved by rapid binding of the protein onto a C4 reverse phase cartridge from Michrom Bioresources (8×1 mm) and subsequent washing and elution. The column and autosampler were pre-equilibrated with 20% (v/v) acetonitrile, 80% H_2_O and 0.1% formic acid (w/v), pH 2.2, prior to sample loading. Immediately following sample (0.5 µl) injection, the solvent composition was changed to 100% acetonitrile, 0.1% formic acid. By using a rapid step gradient and very high flow rates of 600 µl/min, the sample was minimally delayed in the flow path to the mass spectrometer, eluting at approximately 0.4 minutes. The column system was equilibrated at 4°C to minimize back-exchange. Data processing was performed with the Bruker Data Analysis software package, version 4.0. The Maximum Entropy devolution routine was used to perform charge-deconvolution for the spectral range of 700 m/z to 1400 m/z, which encompassed the majority of the observed distribution of protein signal. The deconvoluted spectra were exported to ORGIN software and the centroid masses for Gαi1 were calculated and plotted as a function of time.

### Differential Scanning Calorimetry (DSC)

For DSC analysis, Gαi1•GDP, Ric-8A and Ric-8A:Gαi1[ ] were dialyzed against degassed DSC sample buffer: 25 mM PIPES pH 7.2, 150 mM NaCl and 1 mM TCEP, and additionally for the Gαi1•GDP sample, 20 µM GDP. DSC buffer for Gαi1[ ] contained 20% glycerol (v/v). Immediately before DSC analysis, protein samples were clarified by centrifugation at 14,000 RPM for 10 min in a bench-top Eppendorf microfuge. Protein concentrations after dilution, if required, were determined by least squares fitting of predicted protein extinction coefficients to spectra in the 220–420 nm range measured on a HP diode array instrument. The measured values were 3.6 µM for Gαi1•GDP, 5.9 µM for Ric-8A, 5.1 µM for Ric-8:Gαi1[ ] and 7.9 µM for Gαi1[ ]. DSC measurements were conducted using a Microcal capDSC with autosampler (MicroCal, GE Healthcare). After establishing a thermal history by running water *vs.* water scans, three buffer against water scans were conducted for each sample using the corresponding dialysate solution to obtain the buffer Cp over the experimental temperature range. Following this, two buffer vs protein scans were performed. Protein samples were rescanned once to check for thermal reversibility.

A typical thermal cycle involved cooling the instrument to 20°C after which a 10 min. thermal equilibration was initiated. Following thermal equilibration, scanning of the sample was performed at a scan rate of 1°C/min over a 20°C–70°C range using passive feedback gain mode and a filtering period of 5 seconds. Once the experimental high temperature limit was reached (70°C), the instrument was cooled back to the starting temperature.

Data analysis was performed using Origin 7.0 by first subtracting the last buffer scan from the protein thermal scan. After normalizing the data to the protein concentration, a progressive baseline estimation was performed by calculating the fractional contribution of the native and denatured state to the sample Cp at each point beneath the excess heat capacity function, thus producing a smoothly varying function of temperature [50]. Data presented in Figure 7 were corrected by subtraction of the temperature-dependent change in Cp of the buffer. A weighted average thermal profile for Gαi1[ ] and Ric-8A was computed using the expression Cp^Av^ (T) = w^Ric-8A^Cp^Ric-8A^ (T)+w^Gαi1[ ]^ Cp^Gαi1[ ]^ (T), where w^Ric-8A^ and w^Gαi1[ ]^ are weighting factors for Ric-8A and Gαi1[ ] contributions to the heat capacity at temperature T, and Cp^Ric-8A^ and Cp^Gαi1[ ]^ are the heat capacities measured for free Ric-8A and Gαi1[ ] at that temperature.
